# P-2329. Diversity of Rhinovirus Types in Children During the COVID-19 Pandemic, New Vaccine Surveillance Network, 03/01/20-02/28/21

**DOI:** 10.1093/ofid/ofae631.2481

**Published:** 2025-01-29

**Authors:** Dithi Banerjee, Anjana Sasidharan, Stephanie Gummersheimer, Minati Dhar, Janet A Englund, Eileen J Klein, Mary A Staat, Elizabeth P Schlaudecker, Vasanthi Avadhanula, Leila C Sahni, Natasha B Halasa, Geoffrey A Weinberg, Peter G Szilagyi, John V Williams, Benjamin R Clopper, Heidi L Moline, Mary E Moffatt, Jennifer E Schuster, Rangaraj Selvarangan, Emma Angle

**Affiliations:** Children's Mercy Hospital, Overland Park, Kansas; Childrens Mercy Hospital, Missouri, Kansas; Children's Mercy Hospital, Overland Park, Kansas; Children's Mercy Hospital, Overland Park, Kansas; Seattle Children’s Hospital, Seattle, Washington; University of Washington School of Medicine, Seattle, Washington; Cincinnati Children’s Hospital Medical Center, Cincinnati, Ohio; Cincinnati Children's Hospital Medical Center, Cincinnati, Ohio; Baylor College of Medicine, Houston, Texas; Baylor College of Medicine and Texas Children’s Hospital, Houston, Texas; Vanderbilt University Medical Center, Nashville, TN; University of Rochester School of Medicine & Dentistry, Rochester, NY; UCLA School of Medicine, Agoura Hills, California; University of Pittsburgh, Pittsburgh, Pennsylvania; US Centers for Disease Control & Prevention, Buffalo, New York; Centers for Disease Control and Prevention, Atlanta, Georgia; Children's Mercy Kansas City, University of Missouri Kansas City School of Medicine, Kansas City, Missouri; Children’s Mercy Kansas City, Kansas City, Missouri; Children’s Mercy Kansas City, Kansas City, Missouri; University of Missouri- Kansas City, Kansas City, Missouri

## Abstract

**Background:**

During the COVID-19 pandemic, seasonal respiratory virus detections decreased. However, rhinoviruses (RV) continued to circulate in high numbers. Previous data suggest that RV type circulation is heterogenous and diverse. We characterized the distribution of RV species and the diversity of RV types among children with medically attended acute respiratory infection (ARI) during the first year of the pandemic.

*Spring: Feb to Apr, Summer: May to July, Fall: Aug to Oct, Winter: Nov to Jan.

**Table 1.**
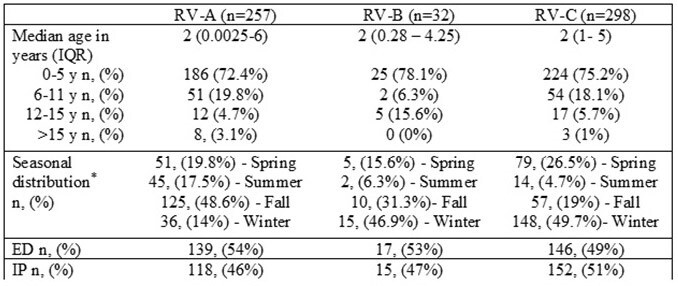
Age, seasonal distribution and ED/hospitalization status based on RV species, New Vaccine Surveillance Network, 03/01/20-02/28/21

**Methods:**

During March 1, 2020 - February 28, 2021, we enrolled children (< 18 years) with ARI seen as either inpatients (IP) or in the emergency department (ED) via prospective surveillance at 7 sites comprising the New Vaccine Surveillance Network. Respiratory specimens were tested for multiple viral pathogens by RT-PCR at each site. A subset of RV/enterovirus (EV) positive specimens were further processed for Sanger sequencing. To obtain a representative subgroup of specimens to sequence within each site, every odd numbered specimen (including co-detections) from ED and IP from every month was selected. Sequenced regions were analyzed using Lasergene software and compared against GenBank sequences using BLAST. Selected demographic data were analyzed by RV species, month, and setting.

**Results:**

Among 4881 enrolled children, RV and/or EV was detected in 1508 (31%). Of these, 675 samples (335 IP and 340 ED) were selected for sequencing based on sampling scheme. Sequencing was successful in 595/675 (88%) extracts including 257 RV-A (43.2%), 32 RV-B (5.4%) and 298 RV-C (50.1%); 7 (1.2%) were EV and 1 was a non-typeable RV. Four of the seven EV samples were reported as EV-D68 on sequencing. Overall, 37 RV-A types, 10 RV-B types and 36 types of RV-C species were identified with A101 (44, 17.1%), B6 (10, 31.1%) and C56 (34, 11.4%) as the most common RV-A, B and C types, respectively. Age, seasonal distribution, and ED/IP status by RV species are described in Table 1 with higher detections reported in children < 5 years.

**Conclusion:**

Among children with medically attended ARI with RV detections, RV-C and RV-A predominated with high type diversity. Most detections were in younger children but occurred similarly in the IP and ED settings. Both species exhibited seasonal trends and high RV diversity that were similar to trends reported in pre-pandemic period.

**Disclosures:**

Janet A. Englund, MD, Abbvie: Advisor/Consultant|AstraZeneca: Advisor/Consultant|AstraZeneca: Grant/Research Support|GlaxoSmithKline: Advisor/Consultant|GlaxoSmithKline: Grant/Research Support|Meissa Vaccines: Advisor/Consultant|Merck: Advisor/Consultant|Pfizer: Board Member|Pfizer: Grant/Research Support|Pfizer: Speaker at meeting|SanofiPasteur: Advisor/Consultant|Shinogi: Advisor/Consultant Mary A. Staat, MD, MPH, Cepheid: Grant/Research Support|Merck: Grant/Research Support|Pfizer: Grant/Research Support|Up-To-Date: Honoraria Elizabeth P. Schlaudecker, MD, MPH, Pfizer: Grant/Research Support|Sanofi Pasteur: Advisor/Consultant Natasha B. Halasa, MD, MPH, Merck: Grant/Research Support Geoffrey A. Weinberg, MD, Inhalon: Advisor/Consultant|Merck & Company: Honoraria for textbook chapter preparation Mary E. Moffatt, M.D., Child Abuse Pediatrics (CAP) representative to Council of Pediatric Subspecialties (CoPS); Current Chair of CoPS Executive Committee: Board Member|I am a volunteer member of the Board of Directors, Kansas Children’s Service League: Board Member|My institution, Children’s Mercy Hospital, Kansas City, receives grant support from CDC for my participation/time dedicated to this research.: Grant/Research Support|My institution, Children’s Mercy Hospital, Kansas City, receives grant support from HRSA for my participation/time dedicated to work on research: Grant/Research Support|My institution, Children’s Mercy Hospital, Kansas City, receives grant support from NIH for my participation/time dedicated to work on research: Grant/Research Support Rangaraj Selvarangan, BVSc, PhD, D(ABMM), FIDSA, FAAM, Abbott: Grant/Research Support|Abbott: Honoraria|BioMerieux: Grant/Research Support|Cepheid: Grant/Research Support|Diasorin: Grant/Research Support|GSK: Advisor/Consultant|Hologic: Grant/Research Support|Luminex: Grant/Research Support|Qiagen: Grant/Research Support

